# EFPA Perspective

**DOI:** 10.1192/j.eurpsy.2021.42

**Published:** 2021-08-13

**Authors:** C. Steinebach

**Affiliations:** Head office, EFPA European Federation of Psychologists’ Associations, Brussels, Belgium

## Abstract

**Introduction:**

Psychologically, the question of profession-specific instruments and tools is not trivial. A profession is characterized by specific knowledge. Knowledge is regarded as part of professional competencies: What is done? How is something done? Why is something done? Knowledge and skills are acquired through specific training and continuing education.

**Objectives:**

Professional knowledge is represented in a specific language. In addition, standards and regulations apply to differentiate it from other professions. Different languages and special professional regulations make cooperation more difficult. These obstacles must be overcome.

**Methods:**

Instruments stand for professional identity. Competence-based tools are subject to professional legal regulations (e.g. following standards defined by EuroPsy Certificate of EFPA), ethical guidelines of the profession (professional ethics according to EFPA Meta-Code of Ethics) and external guidelines for professional practice (e.g. national and EU regulations). This ensures patient safety through Europe-wide standards. The investigation of profession-specific profiles and their modification, also under the conditions of the pandemic, becomes important.

**Results:**

Professional instruments are protected by professional political boundaries. Profession-specific profiles are also an invitation to “coopetition”. While differentiation tends to lead to complementary mission fulfillment in practice, openness leads to a “spill-over of skills” in interdisciplinary practice. Alignment of competence profiles and cooperation are encouraged.

**Conclusion:**

The future certainly lies in closer cooperation between the professions. The search for fundamental common ground (consilience), for effective and sustainable interventions (efficiency) and the demand for evidence-based practice (according to common ethical standards) place the well-founded benefit of an instrument for clients above any other interests.

**Disclosure:**

No significant relationships.

